# Superior oblique myokymia: diagnostic criteria and long-term outcome

**DOI:** 10.1007/s00415-026-13773-6

**Published:** 2026-04-01

**Authors:** Johannes Gerb, Andreas Zwergal, Nicole Lehrer, Nedda Hansel, Marius Näher, Franziska Thiessen, Marianne Dieterich, Doreen Huppert

**Affiliations:** 1 German Center for Vertigo and Balance Disorders, LMU University Hospital, LMU Munich, Munich, Germany; 2 Department of Neurology, LMU University Hospital, LMU Munich, Munich, Germany; 3 Graduate School of Systemic Neurosciences, LMU Munich, Munich, Germany; 4 Munich Cluster for Systems Neurology (SyNergy), Munich, Germany

**Keywords:** Oscillopsia, Vertigo, Visual disorder, Nystagmus, Diplopia, Superior oblique myokymia

## Abstract

**Background:**

Superior oblique myokymia (SOM) is a rare disorder characterized by brief episodes of high-frequency, low-amplitude monocular nystagmus frequently caused by vascular contact with the trochlear nerve. Comprehensive analyses of SOM symptomatology and long-term progression are limited. No diagnostic criteria are available.

**Methods:**

Adult patients who were diagnosed with SOM between 2008 and 2024 were asked to complete a detailed questionnaire on disease symptomatology, time course, and medication. The impact of the disease on patient-reported functioning was assessed using a Likert scale. In addition, medical records were screened in all patients.

**Results:**

A total of 35 patients with SOM were identified (21 females; mean age at symptom onset 42.8 ± 15.0 years). 30 patients experienced visuo-perceptual symptoms (i.e., diplopia or oscillopsia, 85.7%), 22 patients ocular motor symptoms (i.e., noticeable “eye-twitching”, 62.9%), and 15 patients dizziness or gait instability (42.9%). Of 22 patients analyzed for their long-term therapeutic outcome, 13 described symptom improvement with pharmacotherapy, and six without pharmacotherapy. Patients experienced a moderate to high impact of the disease on their daily private and professional lives. Based on these findings, specific diagnostic criteria for SOM were proposed.

**Conclusions:**

SOM can substantially impair patients’ quality of life and daily functioning. The long-term course appears favorable, with most patients experiencing either complete remission or significant symptom reduction over time. Pharmaceutical treatment proved effective in nearly 90% of patients, and spontaneous remission was also observed. Further research should focus on larger patient cohorts defined by clear diagnostic criteria to enhance understanding of SOM’s natural history and treatment outcomes.

## Introduction

**Fig. 1:**
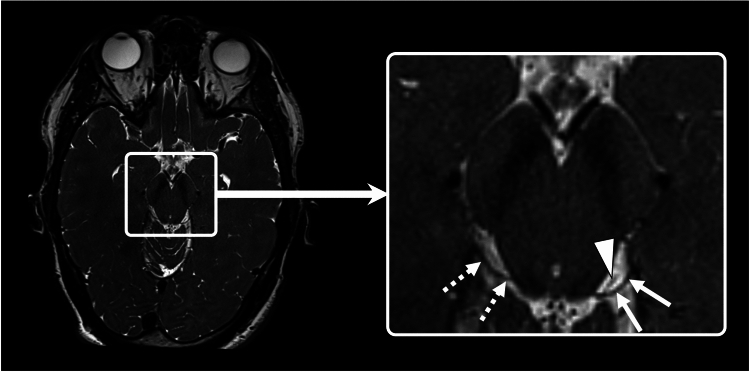
Visualization of the left (solid arrows) and right (dotted arrows) trochlear nerve using constructive interference in steady state (CISS) MRI in a healthy subject. Note how the trochlear nerve exits at the dorsal side of the brainstem. Due to the intensive vasculature of the brainstem, blood vessels are likely to be observable in close proximity to the trochlear nerve (arrowhead); proximity alone does not constitute a functionally relevant nerve compression syndrome

Superior oblique myokymia (SOM) is a rare ocular condition firstly described by Duane in 1906 [1] and termed SOM in 1970 by Hoyt and Keane [2]. It is characterized by episodic, involuntary contractions of the superior oblique muscle resulting in a torsional nystagmus of the affected eye [3]. These contractions lead to transient oscillopsia and diplopia, often causing significant patient discomfort. The pathophysiology of SOM remains poorly understood, though it is thought to involve hyperexcitability of the trochlear nerve, potentially due to a neurovascular conflict [4–7]. Thereby its pathophysiology shares similarities with other presumed vascular nerve compression syndromes such as vestibular paroxysmia (VP) [8, 9] or trigeminal neuralgia [10]. High-resolution magnetic resonance imaging (MRI) can visualise a compression of the trochlear nerve mostly in the ambient cistern by the superior cerebellar artery [5, 11]. However, identification of the trochlear nerve is difficult due to its small dimensions and complex intra-cisternal course [12] and a vascular contact to the trochlear nerve may also be observed in healthy persons (Fig. 1).

Since its initial recognition, SOM has presented a diagnostic challenge due to its intermittent nature, variable clinical presentations and natural course. Historical documentation predominantly focused on case reports and small series, highlighting a need for comprehensive studies investigating the long-term symptom trajectory of this condition. Only two previous case series documented the long-term course of symptoms in SOM [13, 14]. Here, the authors described SOM as a benign, but permanent condition [14] and recommended surgical interventions in patients whose symptoms were refractive to medication [13]. However, these studies were conducted in the 1980 s and 1990 s before the broad availability of current-generation anticonvulsant drugs, and before first reports of the potential therapeutic benefit of topical beta blockers in SOM. Contemporary large-scale updates on SOM therapy and time course based on well-descripted cohorts are lacking.

Individual management of SOM has evolved in the past decades by advancements in both pharmacological and surgical approaches [4], but no standardized treatment guidelines are available. Pharmacological treatments include anticonvulsants, such as carbamazepine and gabapentin [15, 16], which reduce neuronal hyperexcitability. Beta blockers and calcium channel blockers, such as propranolol and verapamil, have also been used with varying degrees of success [17, 18]. For patients with refractory symptoms, surgical options such as tenotomy of the superior oblique tendon [19] or microvascular decompression have been pursued [4, 20]. Despite these interventions, the lack of randomised controlled trials has limited the establishment of standardised treatment protocols, and many patients experience variable symptom control over time.

The current study aims to address critical gaps in knowledge by investigating the long-term course of SOM, including its symptom trajectory, response to treatment, and overall impact on quality of life. By examining a cohort of patients with extended follow-up, this research seeks to provide insights into the long-term evolution of SOM symptoms, and the effectiveness of various therapeutic strategies over time. Furthermore, we strived to propose diagnostic criteria based on commonly reported symptoms and diagnostic findings derived from this patient cohort.

## Methods

### Patients

The databases of the interdisciplinary German Center for Vertigo and Balance Disorders (DSGZ) and the neurological outpatient department at LMU University Hospital, Munich, Germany, were screened for patients presenting with SOM from 2008 to 2024. Medical records were analyzed, including follow-up visits. During the first presentation, all patients had undergone a neurological and neuro-ophthalmological examination including a detailed neuro-orthoptic evaluation by trained neuro-orthopticians (NL). To confirm the diagnosis, a scanning laser ophthalmoscope examination (Rodenstock, Munich, Germany) was performed, along with binocular videooculography (EyeSeeTec, Munich, Germany) to detect monocular nystagmus and to assess hyperventilation-induced aggravation of nystagmus. All patients were initially seen by at least one board-certified neurologist with many years of experience in neuroophthalmology, and in most cases were seen by at least one of the authors.

After approval of the local ethics committee (Number 24–0804), patients were sent the below-described questionnaire, an informed consent form, and a free return envelope via mail. In case of undeliverable letters (e.g., if patients had relocated since their last visit to the hospital), the local registration office was contacted to enquire if more recent addresses of these patients were available. This process is regulated in §§ 44, 45, 46, and 49 of the German Federal Registration Act (Bundesmeldegesetz).

### Questionnaire development

A questionnaire was developed with four categories: symptoms (e.g., frequency of symptoms, impact on private and professional functioning, worsening factors), diagnostic approaches (e.g., time to diagnosis, performed diagnostic tests before diagnosis), therapy (e.g., dosage and effect of medication, time and type of surgery performed), and long-term course. All questions were formulated in simple language (German), and were optimized for clarity and readability by three of the authors (JG, AZ, DH). Answer options were provided as multiple-choice along with space for free text replies. For the impact on daily private and professional functioning, a 5-point Likert scale (ranging from 1: no limitation, to 5: severe limitation of private/professional activities) was used.

### Statistical analysis

The provided answers were further analyzed using Microsoft Excel and JASP (jasp-stats.org). Free text replies were categorised by one of the authors (JG). For numerical data, the mean, minimum, and maximum values as well as the standard deviation were calculated.

## Results

### Cross-sectional patient characteristics at initial diagnosis

**Fig. 2:**
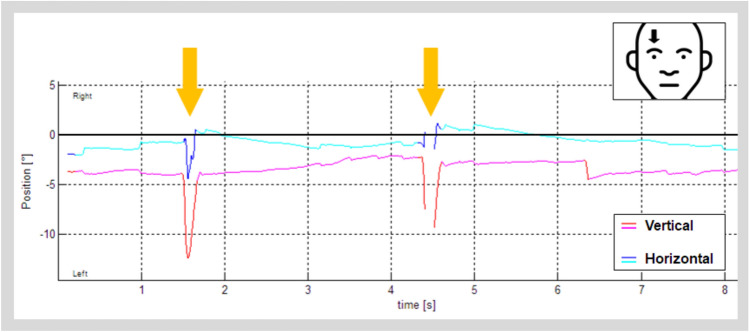
Videooculographic (VOG) recordings of spontaneous eye movements from a patient with right-sided SOM. When the right eye is recorded, two fast bursts of SOM are visible (marked with yellow arrows) at 1.5 s and 4.5 s, respectively. Note that this VOG setting does not detect torsional nystagmus, but instead records the vertical and horizontal components as seen above. Also note that the VOG setup uses algorithmic pupil tracking with an infrared camera, where timepoints without definite pupil detection (e.g., during fast movements, such as in SOM) are automatically discarded, resulting in multiple breaks of the recordings in both plots

35 patients were identified presenting with SOM: 21 females; 21 right-sided SOM, 12 left-sided SOM, 2 unclear side; mean age at symptom onset 42.8 ± 15.0 years, age range 18–70 years, (Table 1). Figure 2 gives an example of monocular nystagmus.

**Table 1:** Demographics and symptomatology of the overall cohort of 35 SOM patients, based on available medical records

	Initial patient cohort
N (of which females)	35 (21 females)
Age	42.8 ± 15.0 years
Side of SOM	21 right-sided, 12 left-sided, 2 patients reporting bilateral symptoms
Reported symptoms	Visual-perceptual symptoms: 30 Ocular motor symptoms: 22 Vestibular–postural symptoms: 15

In order to systematically analyze the symptomatology from the medical records, we divided the symptoms into three conceptually separate categories: visuo-perceptual, ocular motor, and vestibular–postural. In the first category, we aggregated symptoms, which the patient noticed as a change to their visual scene (i.e., diplopia, oscillopsia, blurred vision, reading impairment). The second category included symptoms, which were described as involuntary eye movements such as eye twitches without a subjective perception of a visual disturbance. In the last category, vestibular–perceptive or postural manifestations were collected (i.e., dizziness or gait instability). In total, 30 out of the 35 patients experienced visuo-perceptual symptoms (85.7%), 22 described ocular motor symptoms (62.9%), and 15 vestibular–postural symptoms (42.9%). Importantly, some patients did not describe any visuo-perceptual symptoms at all, but only eye-twitching with (*n* = 1) or without (*n* = 3) additional dizziness or gait instability.

### Patient characteristics at follow-up

**Fig. 3:**
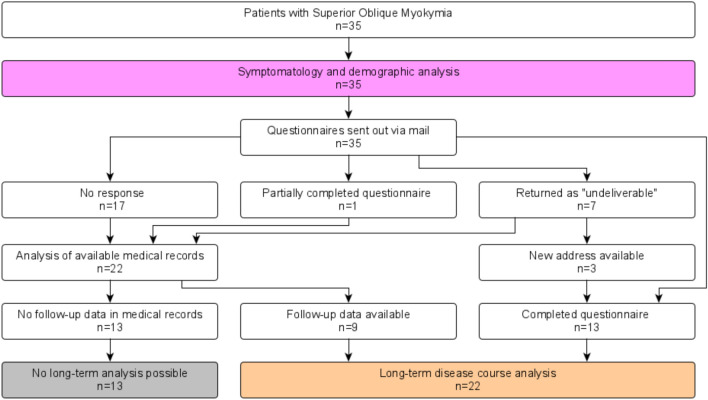
STROBE flow chart of the recruitment process. All 35 SOM patients were analyzed for symptomatology and demographic aspects (pink box). Long-term disease course (orange box) could be analyzed for 22 out of 35 SOM, either as part of the full participation cohort (*n* = 13, i.e., patients who filled out a questionnaire) or in the partial participation cohort (*n* = 9, i.e., patients with sufficient medical records information for retrospective follow-up data analysis). In 13 patients (gray box), no detailed long-term disease course analysis was possible due to a lack of data

All patients were contacted via mail and given the option to participate in the follow-up study. A total of 13 patients provided their informed consent and detailed questionnaire data, while others were lost to follow-up or did not provide complete questionnaires. In some of these patients, follow-up information could be extracted from medical record analysis. An overview of the patient recruitment process can be seen in the STROBE flow chart (Fig. 3).

### Follow-up by questionnaire analysis

From all the patients contacted, 13 patients provided a completely filled out questionnaire (8 females; mean age at symptom onset 42.2 ± 16.2 years, age range 18–70 years). On average, the time between the onset of symptoms and the completion of the questionnaire was 11.7 ± 8.3 years (minimum 5 years, maximum 39 years). Of these patients, 7 described right-sided SOM and 4 left-sided SOM, while the two remaining patients reported binocular symptoms.

#### Epidemiology

The time between symptom onset and SOM diagnosis was less than six months in three patients, less than one year in six patients, and longer than three years in four patients. The SOM diagnosis was conducted by a university hospital (*n* = 8), an ophthalmologist (*n* = 2), or a neurologist (*n* = 1). In two patients, the diagnosis was suggested by either an ophthalmologist (*n* = 1) or a neurologist (*n* = 1), before being confirmed by a university hospital. Eleven of the 13 patients underwent cranial MRI with two MRIs showing neurovascular compression of the trochlear nerve according to the radiological analysis.

#### Symptomatology

**Fig. 4:**
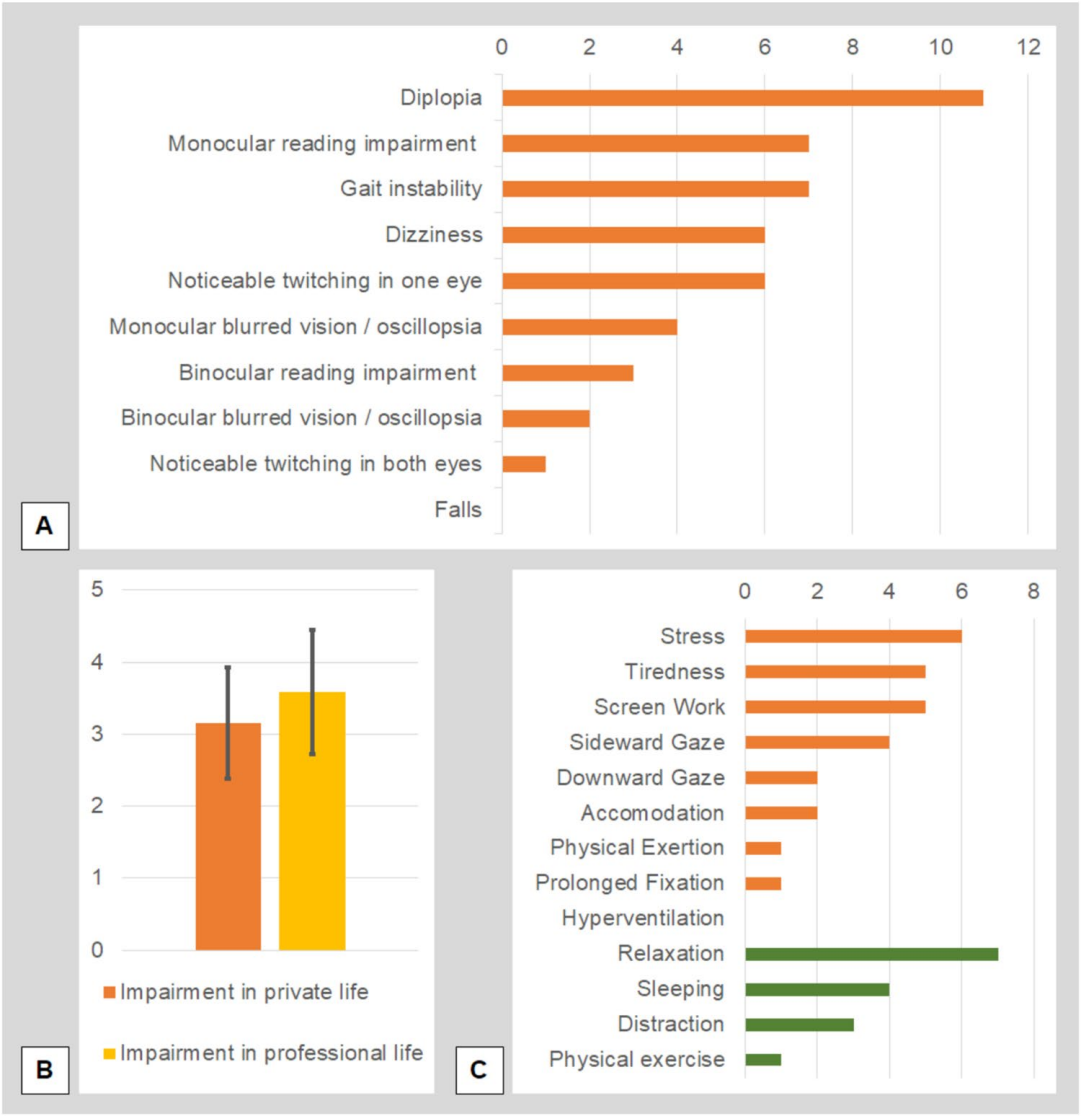
Bar charts of commonly reported symptoms, self-reported impairment, and factors which worsened (orange) or improved (green) symptom severity. **A** Most patients reported diplopia, followed by monocular reading impairment, and gait instability (multiple answers possible, bars indicate the number of patients reporting each option). **B** On average, patients described a moderate to high impairment of their private (orange) and professional (yellow) lives (impairment ranging from 0 (no impairment) to 5 (maximum impairment), self-reported using Likert-scales). **C** Patients typically described symptom worsening (in orange) when stressed, tired, or during screen work, while relaxation, sleeping and distraction commonly mitigated (in green) the symptoms (multiple answers possible, bars indicate the number of patients reporting each option)

All patients reported symptoms from at least one of the three symptom categories (visuo-perceptual, ocular motor, vestibular-postural). One patient described symptoms from two out of the three categories, and nine patients reported symptoms from all three symptom categories. Patients most commonly described diplopia (*n* = 11, 84.6%), gait instability (*n* = 7, 53.8%), reading impairment (unilateral impairment *n* = 7, 53.8%; bilateral impairment *n* = 3, 23.1%), unilateral eye-twitching (*n* = 6, 46.2%), and dizziness (*n* = 6, 46.2%). Blurred vision/oscillopsia was described on one eye by 4 out of the 13 patients (30.8%), and on both eyes by 2 additional patients (15.4%). None of the patients reported falls due to the condition (Fig. 4A).

Seven out of the 13 patients perceived their symptoms as permanent (53.8%), while others reported the typical bursts. Three patients experienced less than 5 symptom bursts per day on average (23.1%), and three patients had more than 5 symptom bursts per day (23.1%).

Patients described a moderate to high disease impact on private functioning (3.2 ± 0.8 points on a 5-point Likert scale, minimum 2, maximum 5) and professional functioning (3.6 ± 0.9 points, minimum 2, maximum 5; Fig. 4B). When asked to provide the reason for this impairment, the most commonly described factors were trouble/inability to drive a car (*n* = 10, 76.9%), or problems when working with screens (*n* = 7, 53.8%). Two patients had problems when communicating with other people, either since eye contact was difficult, or since they did not want other people to notice their condition.

Three patients (23.1%) were on temporary sick leave due to their symptoms, while two additional patients (15.4%) remained on permanent sick leave until an early retirement (ages at symptom onset of these two patients: 58 and 62 years).

The most commonly reported worsening factor was stress (*n* = 6, 46.2%), followed by tiredness (*n* = 5, 38.5%) and working at a screen (*n* = 5, 38.5%). A sideward gaze direction worsened the symptoms in 4/13 patients (30.8%), and downward gaze worsened the symptoms in two patients (*n* = 2, 15.4%). Two patients described problems during accommodation, i.e., when shifting their focus from close objects to more distant objects (*n* = 2, 15.4%). Only one patient described worse symptoms during physical exercise (*n* = 1, 7.7%), and none of the patients reported an effect of heavy breathing/hyperventilation (Fig. 4C).

In most patients, symptom improvement could be achieved by relaxing (*n* = 7, 53.8%) or sleeping (*n* = 4, 30.8%). Other patients indicated that ignoring the bursts until self-limitation was sufficient (*n* = 3, 23.1%, Fig. 4C).

#### Therapy

The most commonly prescribed drug types were anticonvulsants (*n* = 8, 61.5%). Individual patients used over-the-counter eye drops (*n* = 1), homeopathic remedies (*n* = 1), or described an effect of their migraine drugs (topiramate, *n* = 1). Two patients never tried any pharmaceutical intervention. Carbamazepine (CBZ) or oxcarbazepine (OXC) were prescribed to 7 patients, and lacosamide to one patient. Four out of the CBZ/OXC patients were eventually attack free under medication (daily CBZ dosage until attack cessation: 100 mg, 400 mg, 600 mg, 800 mg), while one patient described a reduced attack frequency under a daily CBZ dosage of 1200 mg. One patient had an allergic reaction to OXC, resulting in a hospital stay, and did not continue with the medication. However, this patient described a significantly reduced attack frequency after starting antihypertensive pharmacotherapy with an angiotensin-converting enzyme inhibitor.

One patient was taking various homeopathic remedies consisting of heavily diluted bovine cranial nerve extract and described a substantial frequency reduction under bovine trochlear nerve extract, but not under bovine oculomotor nerve extract.

### Follow-up by medical record analysis

21 patients did not provide the full questionnaire, so medical records were additionally analyzed to determine SOM symptomatology. One patient had developed dementia in the nine years since SOM diagnosis; here, the husband could provide basic information on the efficacy of the prescribed drugs.

Of these 22 patients (14 females, 63.6%), 14 (63.6%) had right-sided SOM. Mean age at symptom onset was 43.2 ± 13.7 years (age range 20–71 years). In 19 out of these 22 patients, medical records allowed for the analysis of time between symptom onset and SOM diagnosis: in six patients this time was less than six months, in three patients less than one year, in five patients less than three years, and more than three years in the remaining five patients.

21 of the 22 patients described visuo-perceptual symptoms (95.5%), nine reported ocular motor symptoms (40.9%) and six reported vestibular–postural symptoms (27.3%). In nine of 22 patients, worsening factors could be derived from the medical records. Here, four patients reported symptom worsening in sideward or downward gaze, three described stress-associated worsening of their symptoms, two experienced worse symptoms in the morning, and two reported worse symptoms during screen work.

Out of the 22 patients, 16 were prescribed carbamazepine (72.7%), once in combination with topical timolol (4.5%), and once in combination with lamotrigine (4.5%); the other prescribed drugs were lacosamide (*n* = 3, 13.6%), oxcarbazepine (*n* = 1, 4.5%), phenytoin (*n* = 1, 4.5%), and topical timolol (*n* = 1, 4.5%).

### Long-term therapeutic outcome

**Fig. 5:**
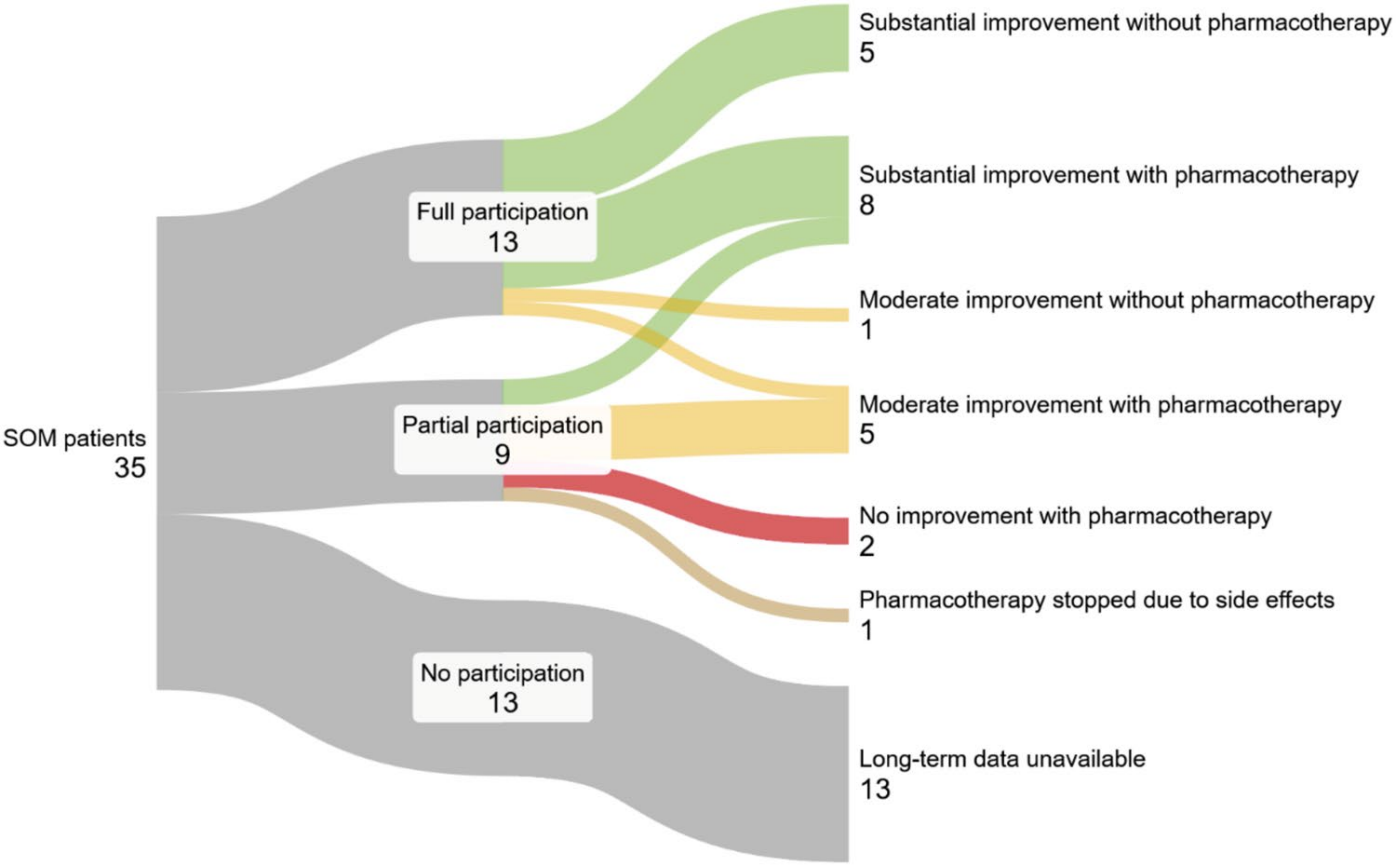
Lankey diagram of the long-term outcome in 35 SOM patients. For 22 patients, the available data allowed for a detailed assessment of therapeutic outcome. Of these, 13 patients described substantial improvement (green), 6 patients a moderate improvement (yellow) and only two patients no effect of pharmacotherapy (red)

In total, 22 patients could be analyzed for their therapeutic outcome (13 based on questionnaire data, nine based on medical records). Out of these, five described substantial symptom improvement without pharmacotherapy, and eight with pharmacotherapy. One patient experienced moderate improvement without pharmacotherapy, and five with pharmacotherapy. Only one patient had stopped pharmacotherapy due to side effects, and only two patients had reported no effect of pharmacotherapy. In the questionnaire-based full participation cohort, the most detailed analyses were possible. After roughly 12 years (time between symptom onset and questionnaire completion: 11.7 ± 8.3 years), only three patients (23.1%) were still taking anticonvulsant medication, while all other patients no longer required pharmacotherapy. Interestingly, 3/13 (23.1%) of patients described recurring episodes of higher disease activity, which were often coinciding with phases of more stress in their life. These episodes were either self-limiting, or patients would resume their respective medication in its last dosage until the episodes ended. An overview of the long-term SOM time course can be seen in Fig. 5.

### Proposed diagnostic criteria

Integrating the findings from detailed questionnaires and medical record analysis in the current study with previously published literature, we propose the following diagnostic criteria for SOM (Table 2). These criteria were designed to include patients who do not subjectively experience visuo-perceptual symptoms; independent of patient reports, an objective quantification of monocular nystagmus is however still mandatory for the diagnosis of definite SOM. In general, the proposed criteria are similar to the current diagnostic criteria for VP [21], with a similar focus on clinical symptoms rather than extensive laboratory testing. Parallel to the diagnostic criteria of VP, treatment response was included in the diagnostic criteria, while detailed eye movement recordings are not necessarily required for SOM diagnosis. Similarly, radiological confirmation of a neurovascular conflict is not part of the proposed diagnostic criteria.

**Table 2:** Proposed diagnostic criteria for definite and probable SOM, with key differences in bold print. Videooculographic or clinical confirmation of a typical SOM nystagmus as well as a treatment response to anticonvulsant medication is mandatory for the diagnosis of definite SOM. In patients where the nystagmus is not observed, e.g., because of self-limitation, only a probable SOM can be diagnosed. Note that not all SOM patients experience visuo-perceptual symptoms. Clinicians should be aware that without detailed nystagmus analysis, i.e., solely based on patient history and treatment response, probable SOM and probable vestibular paroxysmia might be indistinguishable.

Definite SOM	Probable SOM
At least ten episodes with symptoms from at least 2 out of the 3 following symptom categories: - Visuo-perceptual, i.e., diplopia, monocular oscillopsia, or monocular reading impairment - Ocular motor, i.e., a sensation of involuntary monocular eye-twitching/movement - Vestibular-postural, i.e., gait instability or dizziness	
Stereotyped phenomenology in a particular patient	
Duration less than 1 min	**Duration less than 5 min**
Videooculographic or clinical confirmation of monocular vertical–torsional nystagmus, typically increased by hyperventilation, triggered by accommodation, and more pronounced in downward or sideward gaze	**No videooculographic or clinical confirmation of nystagmus/involuntary eye movement**
Response to treatment with anticonvulsant medication (carbamazepine, oxcarbazepine, lacosamide) or topical beta blockers (levobunolol, timolol, betaxolol, propranolol)	Response to treatment with anticonvulsant medication (carbamazepine, oxcarbazepine, lacosamide) or topical beta blockers (levobunolol, timolol, betaxolol, propranolol), or self-limitation
Not better accounted for by other vestibular or neuro-ophthalmological disorder (especially no evidence for vestibular paroxysmia)	

## Discussion

In this detailed evaluation of SOM symptom trajectories, the vast majority of cases showed a benign long-term outcome: almost all patients described substantial or at least moderate symptom improvement, often even without medication. However, during active disease periods, symptoms were not perceived as benign as patients indicated a moderate to high disease impact on their private and professional life, with several patients seeking early retirement due to the severity of symptoms. Many patients had developed their own coping strategies to deal with episodes of increased disease activity, which, in about 25% of patients, were triggered by stress or visual strain. In general, these findings draw a positive picture of the long-term disease course in SOM. However, there is a need for standardised treatment options on the basis of controlled studies. In order to improve comparability of clinical studies, we introduce diagnostic criteria for definite and probable SOM, which should make it possible to overcome the previous diagnostic uncertainties.

Various findings from previous investigations could be confirmed, e.g., the higher rate of right-sided SOM, the average age at symptom onset (typically between 30 and 50 years), as well as a slight female preponderance (in our cohort of 35 patients: 21 females, 60%). This is in line with a previous meta-analysis of all 116 cases published between 1906 and 2018, which had shown a (clinically irrelevant) female preponderance of about 52% [17]. In vestibular paroxysmia, a pathophysiologically similar vascular nerve compression disorder, a small (but again clinically irrelevant) female preponderance of 55% had been found across multiple studies [8]. In trigeminal neuralgia, a similar slight female preponderance of up to 60% has been demonstrated [12, 13]. It is unclear whether this is a sign of anatomical sex differences, e.g., in cranial nerve myelination and subsequent susceptibility to vascular nerve compression [22], or rather a sociodemographic effect of more frequent doctor visits in women [23].

Overall, despite the benign disease course, the impact on daily functioning was substantial. Many patients complained about a lack of knowledge by healthcare professionals, resulting in delayed diagnosis or inappropriate pharmacotherapy. In roughly one-third of patients, a correct diagnosis took more than three years (9/32, 28.1%; no data available from three patients). Fittingly, many patients also explored additional treatment options, which ranged from benign (massages, physiotherapy) to concerning (bovine nerve extract) measures. While drugs of bovine origin have a minuscule, but non-zero chance of contamination and a risk of prion disease [24], these additional treatments are often not covered by insurance companies, and can constitute substantial costs for patients. Especially given the availability of cheap and safe anticonvulsant or topical drugs, this shows how further education about SOM is needed in both patients and healthcare professionals.

Another important aspect of the the long-term SOM course is its episodic nature: in line with previous observations [13, 14], about one quarter of patients experienced intermittent symptom-free intervals followed by (often stress-associated) periods of more pronounced disease activity. This factor should be equally emphasized in patient education, especially because it diverges from the classic doctrine by Rosenberg and Glaser, who in 1983 stated SOM was “benign, but permanent” [14]. In a recent meta-analysis by Zhang et al., the authors stated the wide variability of patient outcomes, “ranging from spontaneous recovery to chronic oscillopsia and diplopia resistant to medical treatment”, but did not describe phases of higher and lower disease activity in detail [17]. Additionally, healthcare providers and clinicians need to be aware of this episodic nature, since patients might present after self-limitation of typical SOM attacks. In this case, only a “probable” SOM should be diagnosed according to our proposed diagnostic criteria.

One interesting aspect of SOM, which we tried to incorporate into the diagnostic criteria, is patients who primarily notice the ocular motor aspects of SOM, i.e., the involuntary monocular eye movements, but do not subjectively experience visuo-perceptual symptoms (no diplopia, no oscillopsia). It appears possible that the lack of oscillopsia might be a compensatory neuronal mechanism, similar to how in physiological fast eye movements (e.g., saccades), perisaccadic visual perception is reduced [25], or how early covert refixation saccades following vestibular impairment are typically not associated with oscillopsia [26]. The lack of oscillopsia may also come from the fact that the amplitude of vertical torsional nystagmus often is small, resulting in only minimal retinal shifts, which can be compensated by the higher-order visual system. Leigh and co-workers reported less than 1° of peak-to-peak torsional and vertical amplitudes [27].

The proposed diagnostic criteria for SOM do purposely not include neuroimaging. This was done in accordance with the diagnostic criteria for VP [21], where radiologically demonstrated neurovascular conflict is not required for diagnosis, since it can commonly be found even in healthy individuals [8]. For the trochlear nerve, there are additional specific difficulties in depicting the nerve’s anatomical course. In our cohort, only two out of eleven patients with available MRI neuroimaging showed a neurovascular contact. However, this analysis was performed on available clinical imaging data, rather than using high-resolution imaging approaches specifically targeted at the analysis of the trochlear nerve. As already demonstrated in VP [9], specialised MR sequences and techniques specifically optimised for the trochlear nerve could have provided better results. Further, it is possible that higher-resolution MRI might be able to demonstrate neurovascular compression in some, if not all, of the SOM patients.

Interestingly, one patient described substantial symptom improvement after beginning antihypertensive treatment with an angiotensin-converting-enzyme inhibitor. With beta blockers (which also have antihypertensive properties) constituting commonly prescribed drugs for SOM [11, 16], this shines a light on how adequate blood pressure management might be important in SOM. It should, however, be noted that while topical beta blockers have also been shown to be effective in SOM [28, 29], no systemic antihypertensive drug effect is assumed in their topical application.

One limitation of the current study lies in the relatively small sample size of detailed questionnaire analyses due to the large number of patients lost to follow-up. This introduces a potential bias in the data. However, given the rarity of SOM, the overall sample size of 35 patients still constitutes one of the largest SOM cohorts to date. In addition, some uncertainty persists regarding the accuracy of patient-reported responses. For instance, certain patients described symptoms as "permanent" without providing further clarification on which specific symptoms they refer to, such as gait instability, diplopia, ocular twitching, or others. They often do not distinguish clearly between ocular symptoms, fear of future attacks, subjective feelings of instability, and other related symptoms. This ambiguity is common in episodic disorders, where patients may initially report a persistent level of disability. Consequently, comprehensive history-taking is essential to differentiate between episodic, transient symptoms and persistent subjective impairments, ensuring accurate diagnosis and appropriate management. As a second limitation with respect to therapeutic options it should be noted that not a single patient had undergone microvascular decompression surgery or ocular muscle tenotomy in the current study. In one previously published case series by Brazis et al., four patients with medication refractive symptoms reported significant improvement following surgery [13]. The same was reported in some case reports [25, 26]. However, undesirable results of surgeries such as diplopia in downward gaze are common [19, 30]. This must be weighed against some studies [17, 18] indicating that patients improved or became symptom-free under medication alone, as in our study. Based on the current data, we cannot provide a clear statement on the role of microvascular surgery as a SOM treatment option. As the vast majority of patients described substantial improvement of symptoms on the longer-term, partially even without medication, one should be cautious with surgery, and consider the potential risks; here, our recommendations are in line with Zhang et al. [17].

Another limitation might be that the questionnaire sent to the patients was text-based, i.e., with written symptom descriptions, although there were multiple options to provide self-written explanations or addendums. Still, more elaborate picture-based questionnaires of subjective visual perception disturbances, which also provide answer options for different types of diplopia or oscillopsia, might allow for easier and more precise assessment of these subjective symptoms [31].

## Conclusion

While there is no standardised treatment for SOM and the disease’s impact on daily life activities might be severe in the acute phase, the long-term course is benign. Pharmacotherapy with anticonvulsants or beta blockers should be offered to the patients, and better education for patients and healthcare professionals is needed. Recurring episodes of increased SOM symptoms are often stress-associated and self-resolving. Surgery might be performed in rare cases of drug intolerance, but the benign long-term course of the disease should be considered.

## Data Availability

Data is available from the corresponding author upon reasonable request.
